# Organizational readiness for knowledge translation in chronic care: a Delphi study

**DOI:** 10.1186/s12913-014-0534-0

**Published:** 2014-11-08

**Authors:** Randa Attieh, Marie-Pierre Gagnon, Carole A Estabrooks, France Légaré, Mathieu Ouimet, Patricia Vazquez, Roberto Nuño

**Affiliations:** Research Centre of the Centre Hospitalier Universitaire de Québec, Hôpital St-François d’Assise, 45 rue Leclerc, Quebec City, QC Canada; Faculty of Nursing, Université Laval, Quebec City, QC Canada; Faculty of Nursing and School of Public Health, University of Alberta, Edmonton, AB Canada; Department of Family Medicine, Université Laval, Quebec City, QC Canada; Department of Political Science, Université Laval, Quebec City, QC Canada; Fundacion Vasca de Innovacion e Investigacion Sanitarias, Bilbao, Spain

**Keywords:** Organizational readiness, Delphi study, Knowledge translation, Chronic care, Measurement

## Abstract

**Background:**

Health-care organizations need to be ready prior to implement evidence-based interventions. In this study, we sought to achieve consensus on a framework to assess the readiness of health-care organizations to implement evidence-based interventions in the context of chronic care.

**Methods:**

We conducted a web-based modified Delphi study between March and May 2013. We contacted 76 potentially eligible international experts working in the fields of organizational readiness (OR), knowledge translation (KT), and chronic care to comment upon the 76 elements resulting from our proposed conceptual map. This conceptual map was based on a systematic review of the existing frameworks of Organizational Readiness for Change (ORC) in health-care. We developed a conceptual map that proposed a set of core concepts and their associated 17 dimensions and 59 sub-dimensions. Experts rated their agreement concerning the applicability and importance of ORC elements on a 5-point Likert scale, where 1 indicates total disagreement and 5 indicates total agreement. Two rounds were needed to get a consensus from the experts. Consensus was *a priori* defined as strong (≥75%) or moderate (60-74%). Simple descriptive statistics was used.

**Results:**

In total, 14 participants completed the first round and 10 completed the two rounds. Panel members reached consensus on the applicability and importance of 6 out of 17 dimensions and 28 out of 59 sub-dimensions to assess OR for KT in the context of chronic care. A strong level of consensus (≥75%) was attained on the *Organizational contextual factors*, *Leadership/participation*, *Organizational support*, and *Motivation* dimensions. The *Organizational climate for change* and *Change content* dimensions reached a moderate consensus (60-74%). Experts also reached consensus on 28 out of 59 sub-dimensions to assess OR for KT. Twenty-one sub-dimensions reached a strong consensus (≥75%) and seven a moderate consensus (60-74%).

**Conclusion:**

This study results provided the most important and applicable dimensions and sub-dimensions for assessing OR-KT in the context of chronic care. They can be used to guide the design of an assessment tool to improve knowledge translation in the field of chronic care.

## Background

Health-care organizations need sufficient high levels of readiness prior to implement evidence-based interventions [1,2]. Organizational readiness for change (ORC) in health-care settings is needed to assess an organization’s readiness to implement change in health-care in general [3,4] and in chronic care in particular [5]. ORC is a broad concept that encompasses other concepts found in the literature, such as organizational capacity [6]. Although there have been several attempts at measuring organizational readiness [7], a limited number of valid and reliable measurement tools to assess the degree of readiness to implement evidence-based change is available [3,8].

In previous work, we reviewed and synthesized the existing evidence on conceptual models/frameworks of ORC as the basis for the development of a comprehensive framework of organizational readiness (OR) for knowledge translation (KT) in the context of chronic care (CC) [6]. The focus was to understand OR components relevant to KT for CC interventions. The context of CC was chosen because the research project aims to contribute to advance conceptual and measurement developments in this particular field. CC services will serve as an exemplar in order to validate the instrument that we aim to develop at the end of this project. The selection of relevant theories and frameworks was thus made on the basis that they would be relevant to KT for CC interventions and eventually, KT for other types of interventions.

The 10 theories, theoretical models and conceptual frameworks that we identified often reflect a narrow view of readiness and omit one or more conceptual elements that are important for a comprehensive assessment of ORC [6]. After graphically analyzing the different components of OR gathered from these 10 theories, theoretical models and conceptual frameworks, the conceptual map allowed us identifying five core concepts common to the operationalization of OR for KT [6]. We also highlighted the relationships between the concepts, dimensions and sub-dimensions included in these models and frameworks. This conceptual map enables us to identify 17 dimensions and 59 sub-dimensions potentially important to consider for assessing OR for KT in health-care organizations [6]. The review and the tentative conceptual map provided a useful overview for researchers interested in ORC in general, and of OR for KT in CC in particular. In this Delphi study, we sought to achieve consensus on a framework to assess the readiness of health-care organizations to implement evidence-based interventions in the context of chronic care.

## Method

### Study design

From the existing consensus methods, we chose the Modified Delphi Technique, which is particularly appropriate where relevant knowledge exists in a given field [9]. The modified Delphi is a systemic and interactive group data collection procedure for obtaining forecasts from a panel of selected independent expert participants; it uses a series of questionnaires delivered in multiple iterations [10,11]. The Modified Delphi Technique relies on expert participants analyzing a future scenario and using their expertise to create nominal data, which are assessed using Likert-type scales producing ordinal or interval data [12]. The number of rounds depends on how quickly a consensus emerges. Theoretically, the process can be continuously iterated until consensus is determined to have been achieved [10]. In the present study, this formal consensus method consisted of two rounds with an international expert panel on ORC elements resulting from the literature review and concept map.

### Participants recruitment and inclusion criteria

A two-round web-based Delphi technique was applied in order to reach consensus among international experts on dimensions and sub-dimensions of a framework to assess OR for KT in CC. Target participants included experts in the fields of organizational readiness, chronic care, and knowledge translation, and were identified from their contribution to the literature in these domains and from our personal contacts. A total of 78 experts were emailed an invitation letter soliciting their participation in the research project. The letter provided a brief outline of the project, its objectives, the expected number of rounds, and anticipated time commitment.

#### Ethical consideration

The Delphi panellists were informed in the invitation letter that their participation in this study was entirely voluntary and they were free to withdraw at any time. By responding to the questionnaire, they implicitly consented to participate in the study. The study was anonymous and no personal information was collected. Ethical approval for the study was obtained from the research ethics committee of the Hospital St-François d’Assise of the CHU de Quebec (approved on March 13, 2013; ethics number B13-04-657).

### Data collection

For both rounds, participants were emailed a link to the web-based questionnaire and were allotted one week to complete the questionnaire. Email reminders to complete the questionnaire were sent after 72 hours to participants who had not yet replied.

### Round 1

The first round of the online Delphi study was conducted in March 2013. Experts were asked to rate the applicability and importance of 17 dimensions (higher-level constructs) and 59 sub-dimensions (lower-level constructs) to assess OR for KT in the context of chronic care on a 5 point-Likert scale, where 1 indicates total disagreement and 5 indicates total agreement. Applicability and importance were assessed as follow: “Is the dimension (sub-dimension) applicable to the assessment of organizational readiness for knowledge translation in chronic care?” and “in the context of organizational readiness for knowledge translation in chronic care, is it important to consider the following dimensions (sub-dimensions)?”. A space was provided to comment upon or suggest changes to the wording used. We analyzed responses to round 1in early April 2013, and designed the round-2 questionnaire based on these results.

### Round 2

Questionnaire formats changed during the two Delphi rounds. Both quantitative and qualitative responses to the first Delphi round were considered in revising the second Delphi questionnaire. Revisions aimed to exclude dimensions that had not achieved consensus and to bring modifications to the wording of dimensions based on experts’ comments. We also provided a definition for all dimensions in the second questionnaire. The second-round online questionnaire was distributed in late April 2013 to the 14 experts who completed the first questionnaire. In this second questionnaire, we asked experts to rate the applicability of 30 sub-dimensions (lower-level constructs) for assessing OR for KT in the context of chronic care that corresponded to the six dimensions retained from round one. Participants were invited to reassess their answers in light of their colleagues’ responses to each item, presented as first round score and level of consensus.

### Data analysis

For the purposes of this study, we defined consensus as a “general agreement of a substantial majority” (>75%) [13]. Consensus was deemed “strong” when applicability and importance of a dimension or sub-dimension were rated as 4 or 5 on the Likert scale by at least 75% of the experts in round 2. Consensus was deemed “moderate” when 60% to 74% of participants agreed on both the applicability and importance of a dimension or sub-dimension. “Partial” consensus was obtained when at least 60% of participants reached consensus on only one aspect (applicability or importance) of dimensions or sub-dimensions. Absence of consensus was determined when less than 60% of participants agreed on the applicability and importance of a core element. For each item, percentile scores and interquartile range were calculated in order to determine where consensus was reached on the Likert scale. Tenth percentile scores indicate the lowest rating on the Likert scale upon which at least 90% of participants agreed, and 25^th^ percentile scores indicate 75% agreement. Interquartile range, calculated as a measure of statistical dispersion, indicates the strength of the consensus [14], where 0 specifies a strong group consensus and 2 indicates dispersed responses. We used SPSS version 21 for data analyses.

An independent researcher analyzed quantitative data from both Delphi rounds. In the first round, data from each open-ended question were reviewed alongside the quantitative data. The feedback report of the first Delphi round presented all the items with the expert agreement scores in percentages. If at least 70% of participants agreed or strongly agreed with a statement about a dimension or sub-dimension, it was considered endorsed. If fewer than 60% of participants agreed or strongly agreed with the statement, it was rejected or modified based on experts’ comments. Since a slight increase in the degree of consensus was obtained in the second round compared to the first, final results confirmed stability in responses [10]. The second-round Delphi data showed much more agreement on statements that reached consensus in the first round, but only two statements could be eliminated after the second round. Hence, we decided that another round would not significantly improve the level of consensus achieved.

## Results

### Characteristics of the participants

**Figure 1:**
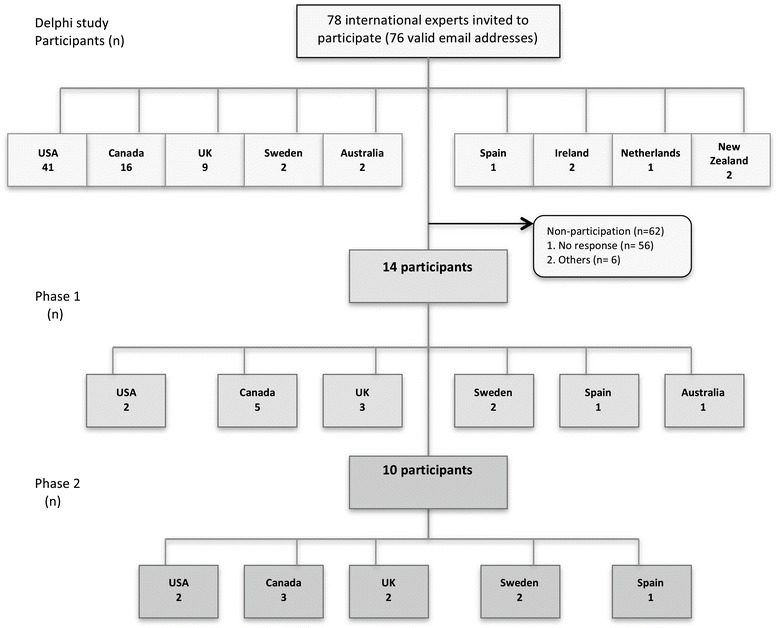
**Flow diagram of the Delphi study participants.**

A total of 76 experts had valid email addresses. Of the 76 persons contacted, 14 experts in the fields of organizational readiness, chronic care, and knowledge translation completed the first data collection round, and 10 the second round. The main reasons for non-participation (82%) were non-response in 90% of cases and others (i.e., expert no longer work in this field, not an expert in this field, no time to do the survey, overcommitted, retirement, personal reasons, out of work, travel, and on vacation) for 10% (Figure 1). The final participants were all mid-career or senior investigators working in universities or governmental organizations. On the 10 final participants, 50% have a combined expertise in organizational readiness (OR), knowledge translation (KT) and chronic care (CC), 30% in KT and CC and 10% in (OR + KT) and (OR + CC) each.

### Results of the first round

Table 1 shows that panel members reached consensus on the applicability and importance of 6 out of 17 dimensions to consider for assessing OR for KT in the context of chronic care. Four dimensions, namely organizational contextual factors, leadership/participation, organizational support, and motivation were considered to be strongly important (≥75%) for the assessment of OR for KT. Two elements – organizational climate for change and change content *–* reached a moderate consensus (60-74%). All these statements were rated 5 on the Likert scale by 70% or more of the panellists.

**Table 1:** **Summary of results for 1** ^**st**^ **round Delphi study**

**Strength of consensus**	**ORKT dimensions & sub-dimensions**		**Criteria**		**5-point Likert score**	**Percentile score**		**Inter-quartile range**
			**A = applicability**	Consensus* (Agreement in %)				
			**I = importance**			**10th**	**25th**	
**Strong**	**Dimension**	**1. Organizational contextual factors**	**A**	**≥75% (85,7%)**	**5**	**2**	**4**	**1**
			**I**	**≥75% (85,7%)**	**5**	**2**	**4**	**1**
Partial	Sub-dimensions	1.1 Healthcare organizational characteristics	A	≥75% (78,6%)	4	3	4	1
			I	<60% (57,2%)	3	3	3	2
Strong		1.2 Organizational culture	A	≥75% (78,6%)	5	3	4	1
			I	≥75% (85,7%)	5	2	4	1
None		1.3 General resources	A	<60% (57,2%)	3	2	3	2
			I	<60% (57,1%)	5	2	3	2
**Moderate**	**Dimension**	**2. Organizational climate for change**	**A**	**≥75% (78,6%)**	**5**	**3**	**4**	**1**
			**I**	**60-74% (71,5%)**	**5**	**3**	**3**	**2**
None	Sub-dimensions	2.1 Mission	A	<60% (50%)	4	1	2	2
			I	<60% (35,7%)	3	1	2	2
None		2.2 Staff cohesion	A	60-74% (64,3%)	4	2	3	1
			I	<60% (57,1%)	4	1	2	2
None		2.3 Autonomy	A	<60% (48,2%)	3	2	3	1
			I	<60% (57,1%)	4	1	2	2
None		2.4 Stress	A	<60% (42,8%)	3	1	3	1
			I	<60% (50%)	5	1	3	2
Strong		2.5 Communication	A	≥75% (85,7%)	5	2	4	1
			I	≥75% (78,6%)	5	2	4	1
Strong		2.6 Openness to change	A	≥75% (100%)	4	4	4	1
			I	≥75% (85,7%)	4	3	4	1
None		2.7 Political change	A	<60% (42,9%)	3	1	3	1
			I	<60% (42,9%)	3	1	2	3
**Strong**	**Dimension**	**3. Management support**	**A**	**≥75% (100%)**	**5**	**4**	**4**	**1**
			**I**	**≥75% (100%)**	**5**	**4**	**4**	**1**
Moderate	Sub-dimensions	3.1 Participation	A	60-74% (71,4%)	4	2	3	2
			I	≥75% (78,6%)	5	2	4	1
Strong		3.2 Leadership / Champion	A	≥75% (92,9%)	4	3	4	1
			I	≥75% (85,7%)	5	3	4	1
**Strong**	**Dimension**	**4. Communication & influence**	**A**	**≥75% (85,7%)**	**4**	**2**	**4**	**1**
			**I**	**≥75% (85,7%)**	**4**	**2**	**4**	**1**
None	Sub-dimensions	4.1 Discussion	A	<60% (50%)	4	1	1	3
			I	<60% (57,2%)	4	1	1	3
None		4.2 Dissemination	A	<60% (50%)	4	1	2	2
			I	<60% (50%)	4	1	2	2
**Strong**	**Dimension**	**5. Motivation**	**A**	**≥75% (92,9%)**	**5**	**3**	**4**	**1**
			**I**	**≥75% (100%)**	**4**	**4**	**4**	**1**
None	Sub-dimensions	5.1 Pressure for change	A	<60% (50%)	3	2	3	1
			I	<60% (57,2%)	4	2	3	2
Moderate		5.2 Change needs	A	60-74% (64,3%)	4	2	3	1
			I	60-74% (64,3%)	4	2	3	1
Moderate		5.3 Training needs	A	≥75% (78,5%)	4	2	4	0
			I	60-74% (64,3%)	4	2	3	2
**Strong**	**Dimension**	**6. Institutional support**	**A**	**≥75% (92,9%)**	**4**	**3**	**4**	**1**
			**I**	**≥75% (85,7%)**	**5**	**3**	**4**	**1**
Strong	Sub-dimensions	6.1 Support climate	A	≥75% (78,6%)	5	1	4	1
			I	≥75% (85,7%)	4	2	4	1
Moderate		6.2 Monitoring	A	60-74% (71,5%)	4	2	3	2
			I	≥75% (85,7%)	4	2	4	1
Strong		6.3 Feedback	A	≥75% (85,8%)	4	3	4	1
			I	≥75% (92,9%)	5	3	4	1
**Strong**	**Dimension**	**7. Human resources**	**A**	**≥75% (85,8%)**	**4**	**2**	**4**	**1**
			**I**	**≥75% (85,8%)**	**4**	**2**	**4**	**1**
Partial	Sub-dimension	7.1 Staff attributes	A	<60% (57,1%)	3	2	3	2
			I	60-70% (64,3%)	4	2	3	1
**Strength of consensus**	**ORKT dimensions & sub-dimensions**		**Criteria**	**Consensus (Agreement in %)**	**5-point Likert score**	**Percentile score**		**Inter-quartile range**
						**10th**	**25th**	
**Moderate**	**Dimension**	**8. Organizational environment readiness**	**A**	**60-74% (71,4%)**	**4**	**2**	**3**	**2**
			**I**	**60-74% (71,4%)**	**4**	**2**	**3**	**2**
None		8.1 Internal turbulences	A	<60% (50%)	3	1	3	1
			I	<60% (35,7%)	3	2	3	1
None	Sub-dimensions	8.2 Intra/inter cooperation	A	<60% (57,2%)	3	3	3	1
			I	<60% (57,2%)	3	3	3	1
None		8.3 Organizational history of innovation	A	<60% (57,1%)	4	2	2	2
			I	<60% (50%)	3	2	3	2
Moderate		8.4 Leader innovativeness	A	60-74% (64,2%)	4	3	3	1
			I	≥75% (78,5%)	4	2	4	0
**Moderate**	**Dimension**	**9. Evidence**	**A**	**60-74% (71,5%)**	**4**	**2**	**3**	**2**
			**I**	**60-74% (64,3%)**	**4**	**2**	**3**	**2**
Moderate		9.1 Patient experiences or preferences	A	60-74% (64,3%)	4	2	3	2
			I	60-74% (71,5%)	4	2	3	2
Moderate	Sub-dimensions	9.2 Research evidence	A	60-74% (71,5%)	4	1	3	2
			I	60-74% (64,3%)	4	1	3	2
Moderate		9.3 Clinical evidence	A	≥75% (78,6%)	4	3	4	1
			I	60-74% (71,5%)	4	3	3	2
**Moderate**	**Dimension**	**10. End-user readiness**	**A**	**≥75% (78,6%)**	**4**	**2**	**4**	**1**
			**I**	**60-74% (64,3%)**	**5**	**2**	**3**	**2**
None	Sub-dimensions	10.1 Background and skills	A	<60% (57,1%)	3	2	3	1
			I	<60% (50%)	3	2	3	1
Strong		10.2 Commitment	A	≥75% (78,6%)	4	3	4	1
			I	≥75% (85,7%)	4	2	4	1
None		10.3 Interpersonal responses to change	A	<60% (57,1%)	3	2	3	1
			I	<60% (57,1%)	4	2	3	1
None		10.4 Desired and perceived involvement	A	<60% (50%)	3	2	3	1
			I	<60% (57,2%)	4	2	3	1
Strong		10.5 Perceptions of benefits	A	≥75% (92,9%)	5	3	4	1
			I	≥75% (92,9%)	5	3	4	1
None		10.6 Satisfaction	A	<60% (35,7%)	3	2	3	1
			I	<60% (35,7%)	3	1	3	2
**Strength of consensus**	**ORKT dimensions & sub-dimensions**		**Criteria**	**Consensus (Agreement in %)**	**5-point Likert score**	**Percentile score**		**Inter-quartile range**
						**10th**	**25th**	
**Partial**	**Dimension**	**11. External influence**	**A**	**60-74% (71,4%)**	**5**	**4**	**4**	**1**
			**I**	**<60% (57,2%)**	**4**	**3**	**3**	**1**
None	Sub-dimensions	11.1 External system	A	<60% (28,6%)	3	2	2	2
			I	<60% (28,5%)	3	2	2	2
None		11.2 Features of the change setting	A	<60% (57,1%)	4	2	3	1
			I	<60% (57,2%)	4	2	3	2
**Partial**	**Dimension**	**12. Operational readiness**	**A**	**60-74% (64,3%)**	**4**	**3**	**3**	**1**
			**I**	**<60% (57,2%)**	**3**	**2**	**3**	**2**
None	Sub-dimensions	12.1 Durability	A	<60% (42,9%)	4	1	2	2
			I	<60% (42,9%)	4	1	2	2
None		12.2 Consistency	A	<60% (28,6%)	3	1	3	1
			I	<60% (35,7%)	3	1	2	2
None		12.3 Reliability	A	<60% (35,7%)	3	1	3	1
			I	<60% (28,5%)	3	1	3	1
Moderate		12.4 Accessibility	A	60-74% (64,3%)	4	1	3	2
			I	60-74% (64,3%)	4	1	3	2
None		12.5 Processing speed	A	<60% (42,8%)	3	1	2	1
			I	<60% (35,7%)	3	1	2	1
Moderate		12.6 Ease to use	A	60-74% (71,4%)	4	2	3	1
			I	60-74% (71,4%)	4	3	3	1
**None**	**Dimension**	**13. Organizational change content**	**A**	**<60% (57,1%)**	**5**	**2**	**3**	**2**
			**I**	**<60% (57,2%)**	**5**	**2**	**3**	**2**
Partial	Sub-dimensions	13.1 Values and goals	A	60-74% (64,3%)	4	3	3	1
			I	<60% (57,2%)	3	2	3	2
Strong		13.2 Attributes of change	A	≥75% (78,6%)	4	3	4	1
			I	≥75% (78,6%)	4	3	4	1
**None**	**Dimension**	**14. Perceived options for change**	**A**	**<60% (57,2%)**	**5**	**2**	**3**	**2**
			**I**	**<60% (57,2%)**	**4**	**2**	**3**	**2**
None	Sub-dimensions	14.1 Understand history of change	A	<60% (50%)	2	1	2	1
			I	<60% (35,7%)	3	1	2	1
Partial		14.2 Approach to change initiatives	A	<60% (57,1%)	4	2	3	1
			I	60-74% (64,3%)	3	3	3	2
None		14.3 Evaluation of opportunities for change	A	<60% (50%)	3	2	3	1
			I	<60% (50%)	3	2	3	1
**Strength of consensus**	**ORKT dimensions & sub-dimensions**		**Criteria**	**Consensus (Agreement in %)**	**5-point Likert score**	**Percentile score**		**Inter-quartile range**
						**10th**	**25th**	
**None**	**Dimension**	**15. Knowledge readiness**	**A**	**<60% (35,7%)**	**3**	**2**	**3**	**2**
			**I**	**<60% (42,9%)**	**3**	**2**	**3**	**2**
None	Sub-dimensions	15.1 General knowledge	A	<60% (35,7%)	3	1	2	2
			I	<60% (35,7%)	3	1	2	2
Moderate		15.2 Specific knowledge	A	60-74% (71,5%)	4	3	3	2
			I	60-74% (71,5%)	4	3	3	2
**None**	**Dimension**	**16. Process readiness**	**A**	**<60% (50%)**	**3**	**2**	**3**	**1**
			**I**	**<60% (42,8%)**	**3**	**2**	**3**	**1**
Moderate	Sub-dimensions	16.1 Evaluation process	A	60-74% (71,4%)	4	3	3	2
			I	60-74% (71,4%)	4	3	3	2
None		16.2 Quality process	A	<60% (57,1%)	4	2	3	1
			I	<60% (50%)	4	2	3	1
None		16.3 Financial management	A	<60% (42,9%)	3	2	3	2
			I	<60% (42,8%)	3	2	3	1
Strong		16.4 Strategic planning process	A	≥75% (78,6%)	4	3	4	1
			I	≥75% (78,6%)	4	3	4	1
Strong		16.5 Decision-making process	A	≥75% (78,6%)	4	3	4	1
			I	≥75% (85,7%)	4	2	4	0
**None**	**Dimension**	**17. Innovation customization process**	**A**	**<60% (42,9%)**	**2**	**2**	**2**	**2**
			**I**	**<60% (42,9%)**	**2**	**2**	**2**	**2**
Partial	Sub-dimensions	17.1 Routine use	A	<60% (57,1%)	4	1	2	2
			I	60-74% (64,3%)	4	1	2	3
Partial		17.2 Adoption consequences	A	60-74% (71,4%)	4	3	3	1
			I	<60% (51,5%)	4	3	3	1
None		17.3 Innovation diffusion	A	<60% (21,4%)	5	1	2	3
			I	<60% (21,4%)	5	1	1	4
Moderate		17.4 Innovation implementation	A	≥75% (85,7%)	4	3	4	0
			I	60-74% (71,5%)	4	3	3	2
Moderate		17.5 Innovation adoption	A	60-74% (64,3%)	5	1	3	2
			I	60-74% (64,3%)	5	1	3	2

A = applicability, I = importance.

*Agreement in %: ≥75% agreement, 60-74% agreement, <60% agreement.

*Bold text in the table highlights the Dimensions.

### Results of the second round

After the first round, almost all sub-dimensions were rated important to assess the implementation of evidence-based practice based on a KT approach in health-care sector. Those that were rated less important with a high agreement were discarded. For this reason, the research team decided to assess only the applicability of the sub-dimensions in the second round Delphi. Table 2 shows the 28 out of 59 sub-dimensions that reached consensus on the applicability of assessing OR for KT in chronic care. Twenty-one sub-dimensions reached a strong consensus (≥75%) and seven a moderate consensus (60-74%).

**Table 2:** **Summary of results for 2** ^**d**^ **round Delphi study**

**Strength of consensus**	**ORKT sub-dimensions**	**Criteria A = applicability**	Consensus* (Agreement in %)	**5-point Likert score**	**Percentile score**		**Inter-quartile range**
					**10th**	**25th**	
	Staff cohesion	A	≥75% (80%)	4	3	4	1
	Staff work-related stress	A	≥75% (90%)	4	3	4	1
**Strong**	Communication about change	A	≥75% (90%)	5	3	4	1
	Manager’s openness to change	A	≥75% (80%)	4	3	4	1
	Appropriate human resources to support change	A	≥75% (100%)	5	4	4	1
	Appropriate material resources to support change	A	≥75% (80%)	5	3	4	1
	Attributes of change	A	≥75% (100%)	4	4	4	1
	Perceived complexity of change	A	≥75% (100%)	4	4	4	1
	Patient experiences and preferences	A	≥75% (80%)	4	3	4	1
	Clinical evidence supporting change	A	≥75% (80%)	4	2	4	1
	Staff participation	A	≥75% (90%)	4	3	4	1
	Leadership/Champion	A	≥75% (100%)	5	4	5	0
	Strategic planning process	A	≥75% (90%)	4	3	4	0
	Adequate level of involvement	A	≥75% (80%)	4	3	4	1
	Evaluation process	A	≥75% (100%)	4	4	4	1
	Feedback	A	≥75% (100%)	5	4	4	1
	Pressure for change	A	≥75% 100%)	4	3	4	0
	Change needs	A	≥75% (90%)	4	3	4	0
	Adequate knowledge and skills	A	≥75% (80%)	4	2	4	0
	Commitment	A	≥75% (90%)	5	2	4	1
	Perception of benefits	A	≥75% (100%)	4	4	4	1
**Moderate**	Change alignment with organization’s mission	A	60-74% (70%)	3	2	3	0
	Organizational culture	A	60-74% (70%)	5	3	3	2
	Research evidence supporting change	A	60-74% (60%)	4	2	3	1
	Leader innovativeness	A	60-74% (60%)	3	3	3	2
	Decision-making process	A	60-74% (70%)	4	3	3	1
	Support climate	A	60-74% (70%)	4	3	3	1
	Monitoring	A	60-74% (60%)	4	2	3	1
	Training and education needs	A	60-74% (70%)	4	2	3	1
**None**	Organizational history of innovation	A	>60% (40%)	3	2	2	2

A = applicability.

*Agreement in %: ≥75% agreement, 60-74% agreement, <60% agreement.

‘Organizational contextual factors’ represent the circumstances under which the change is occurring [7]. This category is linked to two sub-dimensions that were strongly (≥75%) endorsed, namely appropriate human resources to support change and appropriate material resources to support change. It is also linked to organizational culture, although with lesser agreement (60-74%).

‘Leadership/participation’, defined as leadership and collective involvement for change [15,16], is linked to five sub-dimensions, four of which were classified as strongly important (≥75%), namely staff participation, leadership/champion, strategic-planning process and adequate level of involvement. The decision-making process sub-dimension received 70% agreement among experts.

‘Organizational support’, which refers to how the organization sustains and supports change [17], is linked to four sub-dimensions: feedback, evaluation process, monitoring, and support climate. All sub-dimensions reached a strong level of consensus (≥75%) among experts.

‘Motivation’ refers to collective desire or interest with regards to making an effort toward reaching a particular goal [15] and is linked to six sub-dimensions. Panellists agreed that all these sub-dimensions were applicable and important in assessing OR for KT in the context of chronic care. Pressure for change, change needs, adequate knowledge and skills, commitment, and perception of benefits received a strong consensus, with at least 90% agreement. The training and education needs sub-dimension reached a moderate level of consensus (60-74%).

‘Organizational climate for change’, defined as the collective appraisal of the internal organizational environment [18], is related to four sub-dimensions that are applicable and important in assessing OR for KT in the context of chronic care according to panellists. Staff cohesion, staff work-related stress, and communication about change were endorsed with a strong consensus (≥75%). However, change alignment with the organization’s mission received less endorsement (rated as 3 on the Likert scale) by 70% of the experts.

‘Change content’, which refers to the particular change that is being introduced and its characteristics [7], is linked to five sub-dimensions, four of which were endorsed with a strong consensus (≥75%), namely attributes of change, perceived complexity, patient experiences and preferences, and clinical evidence supporting change. The other sub-dimension, evidence-supporting change, reached a moderate level of consensus (60-74%).

## Discussion

This Delphi study identified the most important and applicable dimensions and sub-dimensions for assessing OR for KT in the context of chronic care. Panel members reached consensus on 6 out of 17 dimensions and 17 out of 59 sub-dimensions. These results lead us to four main observations.

First, our findings showed that a consensus was obtained for a total of six dimensions and their related 28 sub-dimensions to assess OR for KT in the context of chronic care. The dimensions represent higher-level theoretical constructs and include the following: organizational contextual factors, leadership/participation, organizational support, motivation, organizational climate for change, and change content. Consistent with the considerable body of literature that suggests that organizational contextual factors influence readiness [7], our findings showed that this dimension is considered key in the assessment of OR for KT. Weiner [19], Holt et al. [7], and Kitson et al. [20] have argued that organizational contextual factors are a necessary dimension of readiness prior to a successful implementation of any change. These contextual factors include elements such as appropriate human and material resources and organizational culture, which involve an understanding of the attributes of the organizational environment within which the change is occurring [7,21]. Further, Weiner [19] argued that the content of change matters as much as the context of change. Change content and the change context are central dimensions of many instruments developed to assess ORC [7]. Consistent with this literature, our study showed that the panel members consented on content of change dimension to consider in the assessment of OR for KT in the context of chronic care. Organizational readiness for change is influenced by *what* is being changed, for example, change attributes, perceived complexity of change, clinical and research evidence, and patient experiences.

Second, according to Pare et al. [22], leadership/participation is likely to be positively associated with organizational readiness for change. Our results support these findings since leadership/participation was strongly endorsed as an important dimension to consider for assessing OR for KT in the context of chronic care. The effective collective involvement of leaders in an organization is crucial for obtaining and sustaining collaborative relationships [23]. Therefore, the leadership/participation dimension is intrinsic to the success of a change [23]. The leadership/participation dimension encompasses building organizational support at all levels [24]. Panellists in this Delphi study identified that establishing staff participation, a strategic-planning process, an adequate level of involvement, a champion to uptake the change, and a decision-making process were all important sub-dimensions related to the leadership/participation dimension.

Third, according to Simpson [17], motivation normally leads to adequate readiness for change. As stated by Wen [25], organizational motivation is one of the most important dimensions in predicting successful implementation of any change. In order to be motivated to support a change, individuals must not only feel that the change is appropriate but also that success is possible [22]. Another dimension that could be relevant in assessing OR for KT in the context of chronic care is organizational support. The latter notion is an important element to assess in the change process, as stated by Lehman [18]. This dimension is achieved through feedback, evaluation process, monitoring, and support climate. In addition, the successful implementation of a given change requires an internal organizational environment that makes the change sustainable [17]. The organizational climate for change dimension, which includes staff cohesion, staff work-related stress, and communication about change, encompasses the organizational dynamics that facilitate an environment of cooperation and trust among staff and information exchange [26].

Fourth, according to the findings of a literature review of ORC measurement instruments that could apply to knowledge translation in health-care [Gagnon M-P, Attieh R, Gandour EK, Légaré F, Ouimet M, Estabrooks CA and Grimshaw J: **A systematic review of instruments to assess organizational readiness for knowledge translation in health care,** forthcoming**]**, there is little improvement in the development of ORC measurement instruments that could be applied to KT in the health-care sector. Given the limited valid and reliable ORC measurement instruments that are adapted or tailored to assess an organization’s readiness prior to implementing evidence-based interventions [[3,7], Gagnon M-P, Attieh R, Gandour EK, Légaré F, Ouimet M, Estabrooks CA and Grimshaw J: **A systematic review of instruments to assess organizational readiness for knowledge translation in health care,** forthcoming], Shea et al. [27] developed and assessed a new theory-based measure called Organizational Readiness for Implementing Change (ORIC) that could help to advance scientific knowledge of the determinants and outcomes of readiness, or provide evidence-based guidance to organizational leaders about how to increase readiness in health-care services [27]. However, as stated by Shea et al. [27], although ORIC shows promise, further psychometric assessment of this tool is needed.

The results from this Delphi study made it possible for a panel of international experts to reach a consensus regarding the dimensions and sub-dimensions that are most important and applicable for assessing OR for KT in the context of chronic care. However, more work is needed to operationalize these constructs and translate them into a measurement instrument for assessing the readiness of health-care organizations to implement evidence-based interventions in chronic care.

### Study limitations

Although this study provides a first step towards the development of a sound instrument for assessing OR for KT in the context of chronic care, some limitations must be acknowledged when considering our findings. First, the small survey sample may reduce the generalizability of results. The response rate for round 1 was low. Only 14 experts responded positively to our invitation. However, retention in the study was good, as 10 out of 14 participants completed both rounds. Reasons provided by non-participants were mostly related to time issues and a perceived level of expertise in the domain. Contacted experts were world-renowned scientists for whom filling out a long online survey is likely to represent an important time investment. The first Delphi questionnaire could be off-putting due to the number of items it contained (78 items), but the second questionnaire was shorter (30 items) and obtained a 71% response rate.

Moreover, the topic of this Delphi study could be perceived as very narrow, and experts in one of the targeted fields (i.e. organizational readiness, knowledge translation or chronic care) could have thought that they were not sufficiently knowledgeable with respect to all dimensions of the topic.

Second, the participants who dropped out of the study and the resulting missing data could represent a bias inflating the degree of consensus. However, only four people dropped out after the first round, and an analysis of their response patterns to the first-round questionnaire showed no difference with other respondents. Finally, the results of this study are based upon the opinions of a limited number of experts who might not represent the views of other scientists in the field. As a lot of work is going on internationally regarding the assessment of organizational readiness for change in health-care organizations [27,28], further theoretical and methodological developments are likely to occur and, it is hoped, will provide more empirical evidence for further consideration of this important dimension for supporting evidence-based innovation in the health-care sphere.

## Conclusion

Although organizational readiness is recognized as a potential facilitator of effective knowledge translation, there is currently a lack of consensus regarding how to assess it. Using a two-round web-based modified Delphi technique, this study sought to achieve consensus among international experts regarding the core elements of a framework to assess OR for KT in the context of chronic care. A consensus was reached for 6 dimensions and 28 sub-dimensions. These results provide a first step towards the identification of the most important and applicable dimensions and sub-dimensions to consider for assessing OR for KT in chronic care. The results could inform the design of an instrument for assessing the readiness of health-care organizations to implement evidence-based interventions in chronic care.

## Abbreviations

CC: Chronic Care
KT: Knowledge translation
ORC: Organizational Readiness for Change
OR: Organizational Readiness
